# Expression of Killer Immunoglobulin Receptor Genes among HIV-Infected Individuals with Non-AIDS Comorbidities

**DOI:** 10.1155/2022/1119611

**Published:** 2022-01-12

**Authors:** Farouk F. Abou Hassan, Mirna Bou Hamdan, Khalil El Asmar, Nada M. Melhem

**Affiliations:** ^1^Medical Laboratory Sciences Program, Division of Health Professions, Faculty of Health Sciences, American University of Beirut, Beirut, Lebanon; ^2^Department of Epidemiology and Population Health, Faculty of Health Sciences, American University of Beirut, Beirut, Lebanon

## Abstract

Combined antiretroviral therapy (cART) increased the life expectancy of people living with HIV (PLHIV) and remarkably reduced the morbidity and mortality associated with HIV infection. However, non-AIDS associated comorbidities including diabetes, hypertension, hyperlipidemia, and cardiovascular diseases (CVD) are increasingly reported among PLHIV receiving cART. Killer cell immunoglobulin receptors (KIRs) expressed on the surface of natural killer (NK) cells have been previously implicated in controlling HIV disease progression. The aim of this study is to investigate the role of KIRs in developing non-AIDS associated comorbidities among PLHIV. Demographic and behavioral data were collected from voluntary participants using a standardized questionnaire. Whole blood samples were collected for KIR genotyping. Hypertension (29.5%) and hyperlipidemia (29.5%) followed by diabetes (23.7%) and CVD (9.7%) were mainly reported among our study participants with higher rate of comorbid conditions observed among participants > 40 years old. The observed KIR frequency (OF) was ≥90% for inhibitory *KIR2DL1* and *KIR3DL1*, activating *KIR2DS4* and the pseudogene *KIR2DP1* among study participants. We detected significant differences in the expression of *KIR3DS4* and *KIR3DL1* (*p* = 0.038) between diabetic and nondiabetic and in the expression of *KIR2DL3* between hypertensive and normotensive HIV-infected individuals (*p* = 0.047). Moreover, *KIR2DL1* and *KIR2DP1* were associated with significantly reduced odds of having CVD (OR 0.08; 95% CI: 0.01-0.69; *p* = 0.022). Our study suggests the potential role of KIR in predisposition to non-AIDS comorbidities among PLHIV and underscores the need for more studies to further elucidate the role of KIRs in this population.

## 1. Introduction

The use of combined antiretroviral therapy (cART) significantly reduced the morbidity and mortality associated with human immunodeficinecy virus (HIV) infection [1]. The former contributed to an increase in the life expectancy of people living with HIV (PLHIV) approaching that of HIV-negative individuals [2–4]. Consequently, the global proportion of people aging with HIV was estimated to reach 21% [5]. These data suggest a demographic shift affecting HIV management and care. With the increasing proportion of people living and aging with HIV, non-AIDS (acquired immunodeficiency syndrome) comorbidities have been increasingly reported among treated PLHIV leading to an increased number of deaths exceeding those of AIDS-related deaths [6–9]. These comorbidities include cardiovascular disease (CVD) [10–15], liver disease [16, 17], renal disease [11, 14, 18], diabetes [10–13, 15, 19], and neurocognitive abnormalities [20, 21], as well as non-AIDS defining malignancies including liver, brain, anal, and lung cancers [22, 23]. While biological aging was suggested to start earlier among HIV infected individuals (55 vs. 65 years) [24], the subsequent pathway leading to disease manifestation among treated and aging PLHIV is not fully understood.

Killer cell immunoglobulin receptors (KIRs) are highly polymorphic type 1 transmembrane glycoproteins expressed on the surface of natural killer (NK) cells [25]. The balance between the inhibitory and activating signals received from KIRs regulates the function of NK cells. Genes encoding KIRs are located in the leukocyte receptor complex (LRC) on chromosome 19q13.4 [26]. There are 16 recognized genes that encode for KIR of which 7 are activating (*KIR2DS1*, *KIR2DS2*, *KIR2DS3*, *KIR2DS4*, *KIR2DS5A*, *KIR2DS5B*, and *KIR3DS1*), 8 are inhibitory (*KIR2DL1*, *KIR2DL2*, *KIR2DL3*, *KIR2DL5A*, *KIR2DL5B*, *KIR3DL1*, *KIR3DL2*, and *KIR3DL3*), and one (*KIR2DL4*) predominantly activating but can transmit inhibitory signals [27]. Two KIR haplotypes are defined in humans: haplotype A and haplotype B [27, 28]. While haplotype A encodes inhibitory receptors (KIR2DL1, KIR2DL3, KIR3DL1, KIR3DL2, KIR3DL3, KIR2DP1, KIR3DP1, and KIR2DL4) and one activating receptor KIR2DS4, haplotype B carries a variety of gene combinations and encodes more activating receptors compared to haplotype A. These include KIR3DL3, KIR2DS2, KIR2DL2, KIR2DL5B (inhibitory) KIR2DS3, KIR2DP1, KIR2DL1, KIR3DP1, KIR2DL4, KIR3DS1, KIR2DL5A (inhibitory), KIR2DS5, KIR2DS1, and KIR3DL2 [29].

Despite the high allelic polymorphism of *KIR* genes, almost all individuals possess the following genes referred to as framework loci: *KIR3DL3*, *KIR3DP1*, *KIR2DL4*, and *KIR3DL2* [27, 28]. Each full-length haplotype is characterized by a centrally located 10–15000 bp region containing a recombination hotspot [30]. The latter subdivides the haplotype into two regions, namely, proximal or centromeric (*Cent*) (5′) and distal or telomeric (*Tel*) (3′) regions which are bound by the framework loci. This recombination hotspot is thus the intermediate region between the centromeric and telomeric regions of the haplotype and bound by the four genes of the framework loci with *KIR3DL3* and *KIR3DP1* defining the centromere and *KIR2DL4* and *KIR3DL2* defining the telomere [31]. A centromeric B haplotype is defined by the presence of at least one of the following: *KIR2DS2*, *KIR2DL2*, *KIR2DL5*, *KIR2DS3*, or *KIR2DS5* whereas a centromeric A haplotype by the sole presence of *KIR2DL3*. A telomeric B haplotype is defined by the presence of at least one of the following: *KIR3DS1*, *KIR2DL5*, *KIR2DS3*, *KIR2DS5*, or *KIR2DS1*; on the other hand, a telomeric A haplotype is defined by the presence of *KIR3DL1* and/or *KIR2DS4*. Finally, a KIR group A genotype is defined by having centromeric A/A and telomeric A/A; other combinations were denoted KIR group B genotypes [32].

There is scarcity of data on the effect of aging on *KIR* expression. However, ample data exist on the association between *KIR* expression and susceptibility, severity, clinical course, or clearance of viral infections (H1N1 2009 influenza, hepatitis C virus, and hepatitis B virus) [33–38] as well as HIV. The proportion of homozygote *KIR3DS1* was higher in HIV-exposed uninfected individuals compared to those with primary HIV infection [39], and higher frequencies of *KIR3DS1* were associated with low viral load [40]. In addition, HIV-exposed individuals who carry homozygote *KIR3DS1* without *KIR3DL1* were characterized by a delayed seroconversion compared to exposed individuals with *KIR3DL1/S1* heterozygous genotype [41].

Few studies reported the association between *KIR* gene polymorphism and susceptibility to type 1 diabetes mellitus (T1DM) [42, 43], hypertension [44], and acute ischemic stroke [45]. *KIR2DL2* was suggested as a susceptibility factor whereas *KIR2DL1* and *KIR2DL5* as protective factors for T1DM. *KIR2DL1* and *KIR2DS1* were linked to a decreased risk of T1DM among Asians but not Caucasians [43]. Moreover, the expression of *KIR2DS5* was suggested as protective against hypertension in a cohort of Chinese Han patients [44]. Higher frequencies of *KIR2DL3*, *KIR2DL5B*, *KIR2DS2*, and *KIR2DS4* were reported among patients with ischemic stroke compared to healthy controls [45]. Similarly, the frequency of *KIR2DL3* and *KIR2DL4* was higher in patients with large artery atherosclerosis compared to controls [45].

The aim of this study is to investigate the relationship between *KIR* genes and non-AIDS comorbid conditions among a cohort of PLHIV in Lebanon. To our knowledge, there are no data on the relationship between *KIR* genes expressed among treated HIV-infected individuals and the risk of developing non-AIDS associated comorbid conditions.

## 2. Methods

### 2.1. Study Design and Ethical Approval

This study was conducted at the American University of Beirut (AUB). Voluntary participants were recruited from three recruitment sites: AUB-Medical Center (AUBMC), Lebanese American University Medical Center-Rizk Hospital (LAUMC-RH), and Soins Infirmiers et Developpement Communautaire (SIDC)—a nongovernmental organization providing health services to HIV-infected individuals. Human subject approval was obtained for this study from the Institutional Review Board (IRB) of AUB and the Lebanese American University (LAU). All participants provided informed consent. A standardized questionnaire was administered to a total of 105 treated adult HIV-infected individuals between November 2018 and December 2019. Demographic and behavioral data including lifestyle, smoking, physical activity, substance use and abuse, coinfections, chronic diseases (cardiovascular disease, hypertension, diabetes, lipid and metabolic disorders, cancer, mental health, and others), first-degree family history (i.e., parents or siblings) of chronic diseases, polypharmacy data, and mental health data were collected.

### 2.2. DNA Extraction and KIR Gene Genotyping

Out of the 105 voluntary participants, 103 HIV-infected individuals provided whole blood samples between November 2018 and December 2019. DNA was extracted using the QIAamp DNA Blood Minikit (Qiagen, Germany) as per manufacturer's instructions. The integrity of the extracted DNA was checked by gel electrophoresis, and its concentration was measured by NanoDrop 2000c (Thermo Fisher Scientific). The purified DNA was stored at -20°C. The Polymerase Chain Reaction- (PCR-) based KIR genotyping Sequence-specific Oligonucleotide Hybridization (SSO) Kit (One Lambda, Thermo Fisher, USA) was used to detect the presence and absence of 16 KIR genes, as per manufacturer's instructions. Briefly, 2 *μ*l of DNA at an adjusted concentration of 20 ng/*μ*l was used along with the primer sets in a total volume of 20 *μ*l per PCR reaction to amplify the alleles. All amplifications were performed using SureCycler 8800 (Agilent Technologies) as per the manufacturer's recommendation: 3-minute denaturation step at 96°C followed by 5 cycles of 96°C, 60°C and 72°C for 20 seconds each; 30 cycles of 96°C for 10 seconds, 60°C for 15 seconds, 72°C for 20 seconds; 1 cycle of final elongation step at 72°C for 10 minutes; and finally hold at 4°C. The presence and absence of the following gene loci and variants were determined: *KIR2DL1*, *KIR2DL2*, *KIR2DL3*, *KIR2DL4*, *KIR2DL5*, *KIR2DS1*, *KIR2DS2*, *KIR2DS3*, *KIR2DS4*, *KIR2DS5*, *KIR3DL1*, *KIR3DL2*, *KIR3DL3, KIR3DS1*, *KIR2DP1*, and *KIR3DP1*. Data were acquired using Luminex LABScan 3D™ and analyzed using HLA Fusion™ Research Software Version 6.0.

### 2.3. Haplotype Classification and KIR Gene Frequencies

KIR haplotypes were defined as previously described [46]. Homozygous A haplotype (AA) was defined by the sole presence of *KIR2DL1*, *KIR2DL3*, *KIR3DL1*, *KIR2DS4*, *KIR2DP1*, *KIR3DP1*, *KIR2DL4*, *KIR3DL2*, and *KIR3DL3* genes. Other KIR combinations were referred to as Bx haplotype (i.e., BB or BA). The frequency of KIR was calculated by direct count of the observed phenotype and referred to as observed frequency (OF). In addition, the estimated KIR gene frequency (KF) for the putative loci was calculated using the following formula: KF = 1 − √(1 − OF) based on the assumption of Hardy-Weinberg equilibrium [47]. We further analyzed participants with Bx genotype based on the expression of different KIR genes combinations. We numbered KIR combinations within the Bx genotype by a number (i.e., Bx1, Bx2, Bx3, etc.) and categorized these into centromeric and telomeric KIR clusters as previously described [30, 46]. The centromeric cluster consists of *KIR2DS2*, *KIR2DL2*, *KIR2DL5B*, and *KIR2DS3* combination, while the telomeric half consists of *KIR3DS1*, *KIR2DL5A*, *KIR2DS5*, and *KIR2DS1* genes. Bx genotype can exhibit centromeric KIR cluster (*KIR2DS2*, *KIR2DL2*, *KIR2DL5B*, and *KIR2DS3* combination), telomeric KIR cluster (*KIR3DS1*, *KIR2DL5A*, *KIR2DS5*, and *KIR2DS1* combination) or both [46]. In the analysis, we included the full length *KIR2DL5* since we were unable to get the data pertinent to the frequency of *KIR2DL5A* and *KIR2DL5B*.

### 2.4. Statistical Analysis

We compared the frequency of expression of *KIR* genes and KIR haplotypes between individuals with and without non-AIDS associated comorbid conditions using Fisher's exact test (FET). We performed clustering analysis to identify *KIR* clusters. We compared the frequency of the latter between individuals with and without comorbidities using FET. We also examined the relationship between *KIR* expression and clusters of *KIR* genes versus the presence or absence of non-AIDS-related comorbidities (specifically diabetes, hypertension, hyperlipidemia, and CVD) using multivariate logistic regression model while adjusting for associated risk factors. We corrected for multiple comparisons for post hoc tests using Bonferroni correction; thus, the calculated alpha was 0.05/12 = 0.004, whereby 12 is the total number of KIR genes included in the analysis. All analyses were conducted using STATA SE 13.0.

## 3. Results

### 3.1. Characteristics of Study Participants

The majority of our participants were 45-59 years (41.9%), males (82.9%) and heterosexuals (52.9%) (Table 1). The majority of our participants contracted HIV-1 and started cART more than 10 years ago. NRTI+NNRTI and NRTI+INSTI were the most commonly used drug regimens (Table 1). Forty-two percent of the study participants reported to have one-to-four non-AIDS associated comorbidity with 29.5%, 29.5%, 9.5%, and 8.6% suffering from hypertension, hyperlipidemia, CVD, and diabetes, respectively (Table 1). Following the same trend, antihypertensive and lipid-lowering agents were the most commonly reported non-cART medications. Our results showed that the frequency of comorbid conditions increases with age among PLHIV; 19% of our participants above 40 years old reported one or two comorbid conditions each followed by 10% and 3% suffering from 3 and 4 conditions, respectively (*Abou Hassan* et al. *submitted manuscript*). Individuals less than 40 years old suffered from less disease conditions with 15% and 3% living with one and 2-3 comorbid conditions, respectively.

### 3.2. KIR Gene and Haplotype Frequencies

We divided the 16 *KIR* genes into 3 groups: inhibitory *KIRs*, activating *KIRs*, and pseudogenes, as previously described [29, 48]. The OF frequency of inhibitory *KIR2DL1* and *KIR3DL1* and the pseudogene *KIR2DP1* were detected in ≥90% among study participants. The OF *KIR2DS4* was 90% while the OF of the remaining activating genes ranged between 34% and 52%. The estimated *KIR* gene frequency followed the same order as the OF data (Table 2). As expected, *KIR2DL4*, *KIR3DL2*, *KIR3DL3*, and *KIR3DP1* were expressed in all individuals. The majority of the study participants (73.8%) carried the Bx haplotype while 26% carried the AA haplotype. We did not detect any significant difference when we compared the frequencies of KIR haplotypes among participants who reported having ≥1 comorbid conditions (*χ*2, *p* = 1) (data not shown). Based on *KIR*-gene content, we identified 18 *KIR*-gene profiles among the study participants: one AA profile and 17 Bx profiles (Table 3). The AA *KIR* profile was predominantly expressed (26%) followed by Bx12 (15.5%) and Bx9 (14.6%). Moreover, the Cent-AA (45.6%) followed by Cent-Bx5 (26.2%) and Bx3 (15.5%) profiles was most commonly detected (Table 4). On the telomeric side, the Tel-AA (53.4%), Tel-Bx2 (27.2%), and Tel-Bx1 (9.7%) were the most common profiles. We also detected higher frequency of Cent-B profiles (58%) than Tel-B profiles (48%) (Table 4).

### 3.3. KIR Genotypes and Non-AIDS Comorbid Conditions

For this analysis and thereafter, we studied the genes with enough variability (*KIR2DL1*, *KIR2DL2*, *KIR2DL3*, *KIR2DL5*, *KIR3DL1*, *KIR2DS1*, *KIR2DS2*, *KIR2DS3*, *KIR2DS4*, *KIR2DS5*, *KIR3DS1*, and *KIR2DP1*). We compared the frequency of *KIR* genes between participants with and without diabetes, hyperlipidemia, CVD, or hypertension. We detected the following significant differences: *KIR3DL1* (inhibitory) and *KIR2DS4* (activating) between diabetic and nondiabetic individuals (FET, *p* = 0.038) and *KIR2DL3* (inhibitory) between hypertensives and normotensives (FET, *p* = 0.047) (Table 5). We also detected a significant difference among hypertensive males expressing *KIR2DL3* (FET, *p* = 0.034) (data not shown). Moreover, the expression of *KIR2DL1* (FET, *p* = 0.043), *KIR2DP1* (FET, *p* = 0.043), and *KIR2DS3* (FET, *p* = 0.049) was significantly different among participants with CVD who were >40 years of age. We detected a borderline significant difference (*p* = 0.053) in the expression of *KIR2DL1* and *KIR2DP1* among males with CVD (data not shown). Moreover, there was no significant difference in the expression of Bx or AA haplotypes among participants with or without any of the conditions above (Table 5). Collectively, these results suggest that *KIR3DL1* and *KIR2DS4* as well as *KIR2DL3* can be associated with diabetes and hypertension, respectively, among people living and aging with HIV. These data also suggest that *KIR2DL1*, *KIR2DP1*, and *KIR2DS3* are significantly associated with CVD among participants > 40 years in our cohort.

We then analyzed in a multivariate analysis the relationship between *KIR* genes expression and the risk of having non-AIDS comorbidities. We adjusted for age, sex, and family history for the respective comorbid condition; for hypertension and CVD, we also adjusted for additional risk factors, specifically smoking and alcohol use. Our results showed that participants expressing *KIR2DL1* and *KIR2DP1* were significantly less likely to have CVD (OR 0.08; 95% CI, 0.01-0.69; *p* = 0.022) (Table 6). However, we did not detect any significant difference following post hoc comparisons.

### 3.4. KIR Clusters and Comorbidities

**Figure 1:**
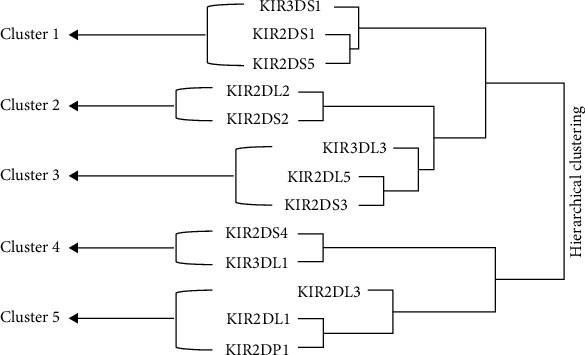
Clustering analysis of KIR genes among study participants.

Based on the *KIR* genes clustering analysis, we identified five *KIR* clusters: Cluster 1, Cluster 2, Cluster 3, Cluster 4, and Cluster 5 (Figure 1). Cluster 1 corresponds to the telomeric cluster containing *KIR3DS1*, *KIR2DS1*, and *KIR2DS5*. We detected significant difference in the expression of Cluster 4 between diabetic and non-diabetic participants (*p* = 0.038) and in the expression of Cluster 5 between hypertensives and normotensives (*p* = 0.047) (Table 7). We did not detect any significant difference between centromeric (Cent-A and Cent-B) and telomeric (Tel-A and Tel-B) clusters and any comorbid condition (data not shown). The multivariate analysis revealed that individuals expressing Cluster 5 have significantly reduced odds of hypertension (OR 0.08; 95% CI: 0.006-0.99; *p* = 0.05) (Table 8).

## 4. Discussion

PLHIV are at higher risk of developing non-AIDS associated comorbidities than the general population; moreover, the prevalence of these comorbidities among PLHIV increases with age [6, 8, 11]. Our data showed that the frequency of comorbid conditions was higher among HIV-positive individuals > 40 years compared to those ≤40 years of age (*Abou Hassan et al. submitted manuscript*). In accordance with previous reports from our group (*Hammad et al. accepted manuscript JIDC*) and worldwide [14, 49–52], the most commonly reported comorbidities among our study participants were hypertension and hyperlipidemia followed by CVD and diabetes.

A limited number of studies exists on the prevalence of chronic disease conditions in the Lebanese population. Recently, the prevalence of hypertension among Lebanese adults (*n* = 2014) was reported at 31%. The former was higher among older participants and those with higher body mass index (BMI) or reported to have CVD [53]. The prevalence of diabetes mellitus in Lebanon (*n* = 17,832) was reported at 7.95%. The prevalence of type 1 diabetes was estimated at 0.1% [54]. Studies on the prevalence of comorbidities among PLHIV in the Middle East and North Africa (MENA) region are currently lacking except for reports from Iran [55] whereby hyperglycemia was recently reported to be highly prevalent among a cohort of PLHIV. This study identified older age, male gender, higher BMI, and prolonged duration of HIV infection as associated risk factors [55]. Recently, diabetes mellitus followed by dyslipidemia and hypertension was predominantly reported among a small group of HIV-infected individuals in western Saudi Arabia with age being a major risk factor [56]. Consequently, more studies are needed to determine the prevalence of non-AIDS associated comorbid conditions among PLHIV in the MENA region.

We observed high frequency of *KIR2DS4*, *KIR2DL1*, *KIR2DP1*, and *KIR3DL1* genes among our study participants. Our results were similar to previously reported data in the region specifically among healthy Lebanese [48, 57], Iranian [58, 59], and Turkish adults [60]. Similar results were also reported in Southern Brazil [61, 62] and Eastern Han populations in China [63]. Moreover, the AA genotype profile was the most frequent among our study participants similar to previously reported data in healthy unrelated individuals in Lebanon [57], Turkey [60], Iran [58], and Tunisia [64].

Several studies reported on *KIR* genes expressions among individuals with chronic conditions such as diabetes [42], hypertension [44], malignancies (colorectal cancer [65], biliary cancer [32], breast cancer [66], and leukemia [67, 68]), and primary immunodeficiency disorders such as common variable immune deficiency (CVID) [69]. While the frequency of *KIR* genes did not differ between diabetics and healthy controls among the Chinese Han [70], Basque [71], and Saudi populations [72], the expression of *KIR2DL3* was significantly different among British HIV-naïve children (<5 years) with type 1 diabetes compared to healthy controls [73]. We report a significant difference in the frequency of *KIR2DS4* and *KIR3DL1* between diabetic and non-diabetic HIV-positive participants. A recent meta-analysis showed that the expressions of *KIR2DL1*, *KIR2DL2*, and *KIR2DL5* were significantly associated with susceptibility to T1DM [42]. *KIR2DL2* was associated with increased risk of type 1 diabetes whereas *KIR2DL1* and *KIR2DL5* decreased the risk of the latter. However, we did not detect any significant difference in the expression of these genes between diabetics and non-diabetics among our study participants. While our results showed a significant difference in the frequency of *KIR2DL3* between hypertensive and normotensive HIV-positive individuals, Wang et al. reported a significant association between the expression of *KIR2DS5* and decreased risk of hypertension [44]. While our data did not reveal any association between the expression of *KIR* genes and risk of non-AIDS comorbidities, this could be due to ethnicity, sample size, and the population under study. Previous studies compared the frequency of *KIR* genes between individuals with a chronic condition and healthy subjects, while we compared the expression of *KIR* genes among HIV-infected subjects with and without non-AIDS comorbid conditions.

Despite the increased expression of centromeric and telomeric *KIR* clusters among hypertensive compared to normotensive participants, there was no significant association between these clusters and non-AIDs comorbid conditions. This is probably due to our small sample size. A recent study in China showed that centromeric KIR cluster (*KIR2DS2-2DL2-2DS3-2DL5*) was significantly increased in children with B-cell acute lymphoblastic leukemia (B-ALL) compared to healthy controls and provided the first evidence that this gene cluster might increase the susceptibility to B-ALL in Chinese Han children [74]. This result suggests that *KIR* gene clusters might be a predisposing factor for susceptibility to diseases and warrants further investigation. To our knowledge, the relationship between *KIR* clusters among PLHIV and non-AIDS comorbid conditions was not previously explored.

Our study has several limitations. Our study lacks a control group of HIV-negative individuals; thus, we were unable to compare the frequencies of *KIR* genes between our cohort and HIV-naïve individuals. Our study is a cross-sectional study without historic clinical and medical data to assess the evolution of comorbid conditions and pertinent risk factors across time. Moreover, we were unable to investigate the interaction between KIRs and their putative human leukocyte antigen class I (HLA-I) ligand. The latter has been implicated in controlling HIV-disease progression [75–79] and in the control or the progression of other viral diseases including human influenza virus, viral hepatitis (HCV and HBV), and human cytomegalovirus (HCMV) [26, 77].

## 5. Conclusion

Additional studies with larger populations are needed to elucidate the role of *KIRs* in susceptibility or resistance to non-AIDS comorbid conditions among PLHIV. Importantly, the burden of non-AIDS comorbidities among people living and aging with HIV is critically needed in the region. These studies are important for the proper management and care of comorbid conditions among PLHIV. Understanding the molecular mechanisms governing the genetic factors modulating living and aging with HIV should be prioritized for screening and intervention to prevent and mitigate multimorbidities among people living and aging with HIV.

## Figures and Tables

**Table 1:** Demographic and clinical characteristics of study participants.

	*n*	%
Gender (*n* = 105)		
Male	87	82.9
Female	18	17.1
Age in years (*n* = 105)		
25-44	42	40
45-59	44	41.9
≥ 60	19	18.1
Sexual history (*n* = 102)		
Heterosexual	54	52.9
MSM	33	32.4
Bisexual	14	13.7
HIV route of transmission (*n* = 105)		
Unprotected sex	72	68.6
Shared needle/syringe	7	6.7
Others	7	6.7
Duration of HIV infection (*n* = 103)		
< 5 years	9	10.3
5-10 years	40	38.8
> 10 years	54	52.4
Duration of cART (*n* = 105)		
< 5 years	16	15.2
5-10 years	42	40
> 10 years	47	44.8
cART regimen (*n* = 102)		
NRTI + NNRTI	47	46.1
NRTI + INSTI	47	46.1
Others (PI + NRTI and/or NNRTI)	8	7.8
≥ 1 non-AIDS associated comorbidities (*n* = 105)	44	41.9
Hypertension	31	29.5
Hyperlipidemia	31	29.5
CVD	10	9.5
Diabetes	9	8.6
Non-cART medication (*n* = 105)		
Antihypertensives	31	29.5
Lipid-lowering agents	31	29.5
Hypoglycaemic agents	9	8.6

MSM: men exclusively having sex with men; cART: combined antiretroviral therapy; NRTI: nucleoside/nucleotide reverse-transcriptase inhibitor; NNRTI: nonnucleoside reverse-transcriptase inhibitor; NNRTI: non-nucleoside reverse transcriptase inhibitor; PI: protease inhibitor.

**Table 2:** The observed and estimated KIR gene frequencies among study participants.

	Inhibitory *KIR*								Activating *KIR*						Pseudogene	
	2DL1	2DL2	2DL3	2DL4	2DL5	3DL1	3DL2	3DL3	2DS1	2DS2	2DS3	2DS4	2DS5	3DS1	2DP1	3DP1
OF	95	52.4	89	100	64	90	100	100	44.7	52.4	34	90	40	43.7	95	100
KLF	0.78	0.31	0.67	1	0.4	0.68	1	1	0.26	0.31	0.19	0.68	0.23	0.25	0.78	1

KIR: killer cell immunoglobulin receptor; OF: observed frequency calculated by direct counting; KLF: estimated KIR gene frequency calculated using the formula 1 − √(1 − OF).

**Table 3:** Distribution of KIR-gene profiles among study participants.

Genotypes	2DL4	3DL1	2DS4	3DS1	2DS5	2DS1	3DL2	2DL3	2DS2	2DL2	2DS3	3DL3	2DP1	2DL1	3DP1	*N*	% (*N* = 103)
AA	*2DL4*	*3DL1*	*2DS4*				*3DL2*	*2DL3*				*3DL3*	*2DP1*	*2DL1*	*3DP1*	27	26.21
Bx1	*2DL4*			*3DS1*	*2DS5*	*2DS1*	*3DL2*		*2DS2*	*2DL2*	*2DS3*	*3DL3*	*2DP1*	*2DL1*	*3DP1*	2	1.94
Bx2	*2DL4*			*3DS1*	*2DS5*	*2DS1*	*3DL2*		*2DS2*	*2DL2*		*3DL3*			*3DP1*	1	0.97
Bx3	*2DL4*	*3DL1*	*2DS4*				*3DL2*	*2DL3*	*2DS2*	*2DL2*		*3DL3*	*2DP1*	*2DL1*	*3DP1*	8	7.77
Bx4	*2DL4*	*3DL1*	*2DS4*	*3DS1*	*2DS5*	*2DS1*	*3DL2*	*2DL3*	*2DS2*	*2DL2*		*3DL3*	*2DP1*	*2DL1*	*3DP1*	7	6.8
Bx5	*2DL4*	*3DL1*	*2DS4*		*2DS5*	*2DS1*	*3DL2*		*2DS2*	*2DL2*		*3DL3*			*3DP1*	2	1.94
Bx6	*2DL4*			*3DS1*	*2DS5*	*2DS1*	*3DL2*	*2DL3*				*3DL3*	*2DP1*	*2DL1*	*3DP1*	4	3.88
Bx7	*2DL4*	*3DL1*	*2DS4*	*3DS1*		*2DS1*	*3DL2*	*2DL3*	*2DS2*	*2DL2*	*2DS3*	*3DL3*	*2DP1*	*2DL1*	*3DP1*	2	1.94
Bx8	*2DL4*	*3DL1*	*2DS4*	*3DS1*	*2DS5*	*2DS1*	*3DL2*	*2DL3*	*2DS2*	*2DL2*	*2DS3*	*3DL3*	*2DP1*	*2DL1*	*3DP1*	5	4.85
Bx9	*2DL4*	*3DL1*	*2DS4*				*3DL2*	*2DL3*	*2DS2*	*2DL2*	*2DS3*	*3DL3*	*2DP1*	*2DL1*	*3DP1*	15	14.56
Bx10	*2DL4*	*3DL1*	*2DS4*				*3DL2*		*2DS2*	*2DL2*	*2DS3*	*3DL3*	*2DP1*	*2DL1*	*3DP1*	3	2.91
Bx11	*2DL4*	*3DL1*	*2DS4*	*3DS1*		*2DS1*	*3DL2*	*2DL3*			*2DS3*	*3DL3*	*2DP1*	*2DL1*	*3DP1*	2	1.94
Bx12	*2DL4*	*3DL1*	*2DS4*	*3DS1*	*2DS5*	*2DS1*	*3DL2*	*2DL3*				*3DL3*	*2DP1*	*2DL1*	*3DP1*	16	15.53
Bx13	*2DL4*			*3DS1*	*2DS5*	*2DS1*	*3DL2*	*2DL3*	*2DS2*	*2DL2*	*2DS3*	*3DL3*	*2DP1*	*2DL1*	*3DP1*	3	2.91
Bx14	*2DL4*	*3DL1*	*2DS4*				*3DL2*		*2DS2*	*2DL2*		*3DL3*			*3DP1*	2	1.94
Bx15	*2DL4*	*3DL1*	*2DS4*	*3DS1*			*3DL2*	*2DL3*	*2DS2*	*2DL2*	*2DS3*	*3DL3*	*2DP1*	*2DL1*	*3DP1*	2	1.94
Bx16	*2DL4*	*3DL1*	*2DS4*	*3DS1*		*2DS1*	*3DL2*		*2DS2*	*2DL2*	*2DS3*	*3DL3*	*2DP1*	*2DL1*	*3DP1*	1	0.97
Bx17	*2DL4*	*3DL1*	*2DS4*		*2DS5*	*2DS1*	*3DL2*	*2DL3*	*2DS2*	*2DL2*		*3DL3*	*2DP1*	*2DL1*	*3DP1*	1	0.97

Gene presence or absence is represented by italic and empty boxes, respectively. Each number next to the genotype represents different KIR combination.

**(a):** 

Centromeric										
Genotype	KIR2DL3	KIR2DS2	KIR2DL2	KIR2DS3	KIR3DL3	KIR2DP1	KIR2DL1	KIR3DP1	*N*	% (*N* = 103)
AA	*KIR2DL3*				*KIR3DL3*	*KIR2DP1*	*KIR2DL1*	*KIR3DP1*	47	45.63
Bx1		*KIR2DS2*	*KIR2DL2*	*KIR2DS3*	*KIR3DL3*	*KIR2DP1*	*KIR2DL1*	*KIR3DP1*	6	5.82
Bx2		*KIR2DS2*	*KIR2DL2*		*KIR3DL3*			*KIR3DP1*	1	0.97
Bx3	*KIR2DL3*	*KIR2DS2*	*KIR2DL2*		*KIR3DL3*	*KIR2DP1*	*KIR2DL1*	*KIR3DP1*	16	15.53
Bx4		*KIR2DS2*	*KIR2DL2*		*KIR3DL3*			*KIR3DP1*	4	3.88
Bx5	*KIR2DL3*	*KIR2DS2*	*KIR2DL2*	*KIR2DS3*	*KIR3DL3*	*KIR2DP1*	*KIR2DL1*	*KIR3DP1*	27	26.21
Bx6	*KIR2DL3*			*KIR2DS3*	*KIR3DL3*	*KIR2DP1*	*KIR2DL1*	*KIR3DP1*	2	1.94

**(b):** 

Telomeric									
Genotype	KIR2DL4	KIR3DL1	KIR2DS4	KIR3DS1	KIR2DS5	KIR2DS1	KIR3DL2	*N*	% (*N* = 103)
AA	*KIR2DL4*	*KIR3DL1*	*KIR2DS4*				*KIR3DL2*	55	53.4
Bx1	*KIR2DL4*			*KIR3DS1*	*KIR2DS5*	*KIR2DS1*	*KIR3DL2*	10	9.70
Bx2	*KIR2DL4*	*KIR3DL1*	*KIR2DS4*	*KIR3DS1*	*KIR2DS5*	*KIR2DS1*	*KIR3DL2*	28	27.18
Bx3	*KIR2DL4*	*KIR3DL1*	*KIR2DS4*		*KIR2DS5*	*KIR2DS1*	*KIR3DL2*	3	2.91
Bx4	*KIR2DL4*	*KIR3DL1*	*KIR2DS4*	*KIR3DS1*		*KIR2DS1*	*KIR3DL2*	3	2.91
Bx5	*KIR2DL4*	*KIR3DL1*	*KIR2DS4*	*KIR3DS1*		*KIR2DS1*	*KIR3DL2*	2	1.94
Bx6	*KIR2DL4*	*KIR3DL1*	*KIR2DS4*	*KIR3DS1*			*KIR3DL2*	2	1.94

Gene presence or absence is represented by italic and empty boxes, respectively. Each number next to the genotype represents different KIR combination.

**Table 5:** Observed frequency of KIR expression among HIV-infected individuals with and without comorbid conditions.

	With diabetes (*N* = 9)	Without diabetes (*N* = 28)	*p* value	With hyperlipidemia (*N* = 31)	Without hyperlipidemia (*N* = 45)	*p* value	With CVD (*N* = 10)	Without CVD (*N* = 92)	*p* value	With hypertension (*N* = 31)	Without hypertension (*N* = 34)	*p* value
*KIR* genes												
KIR2DL1	9 (25%)	27 (75%)	1	29 (39.7%)	44 (60.3%)	0.563	8 (8.3%)	89 (91.7%)	0.074	27 (45%)	33 (55%)	0.184
KIR2DL2	5 (25%)	15 (75%)	1	14 (35%)	26 (65%)	0.352	5 (9.4%)	48 (90.6%)	1	17 (53.1%)	15 (46.9%)	0.46
KIR2DL3	9 (26.5%)	25 (73.5%)	0.562	28 (41.2%)	40 (58.8%)	1	8 (8.8%)	83 (91.2%)	0.294	25 (43.1%)	33 (56.9%)	*0.047* ^∗^
KIR2DL5	7 (28%)	18 (72%)	0.687	20 (39.2%)	31 (60.8%)	0.805	5 (7.7%)	60 (92.3%)	0.49	21 (48.8%)	22 (51.2%)	1
KIR2DP1	9 (25%)	27 (75%)	1	29 (39.7%)	44 (60.3%)	0.563	8 (8.3%)	89 (91.7%)	0.074	27 (45%)	33 (55%)	0.184
KIR2DS1	6 (31.6%)	13 (68.4%)	0.447	16 (42.1%)	22 (57.9%)	1	4 (8.9%)	41 (91.1%)	1	17 (53.1%)	15 (46.9%)	0.46
KIR2DS2	5 (25%)	15 (75%)	1	14 (35%)	26 (65%)	0.352	5 (9.4%)	48 (90.6%)	1	17 (53.1%)	15 (46.9%)	0.46
KIR2DS3	4 (33.3%)	8 (66.7%)	0.432	11 (42.3%)	15 (57.7%)	1	1 (2.9%)	33 (97.1%)	0.159	11 (55%)	9 (45%)	0.591
KIR2DS4	6 (18.2%)	27 (81.8%)	*0.038* ^∗^	26 (38.2%)	42 (61.8%)	0.259	10 (10.9%)	82 (89.1%)	0.592	26 (45.6%)	31 (54.4%)	0.463
KIR2DS5	6 (33.3%)	12 (66.7%)	0.269	13 (39.4%)	20 (60.6%)	1	4 (10%)	36 (90%)	1	15 (51.7%)	14 (48.3%)	0.622
KIR3DL1	6 (18.2%)	27 (81.8%)	*0.038* ^∗^	26 (38.2%)	42 (61.8%)	0.259	10 (10.9%)	82 (89.1%)	0.592	26 (45.6%)	31 (54.4%)	0.463
KIR3DS1	6 (35.3%)	11 (64.7%)	0.251	16 (44.4%)	20 (55.6%)	0.642	3 (6.8%)	41 (93.2%)	0.508	16 (55.2%)	13 (44.8%)	0.324
*KIR* haplotypes												
AA	1 (10%)	9 (90%)	0.393	8 (47.1%)	9 (52.9%)	0.585	3 (11.1%)	24 (88.9%)	0.722	7 (39%)	11 (61%)	0.418
Bx	8 (29.6%)	19 (70.4)		23 (39%)	36 (61%)		7 (9.3%)	68 (90.2%)		24 (51.1%)	23 (48.9%)	

CVD: cardiovascular disease. Significant *p* values (*p* < 0.05) of Fisher's exact test are indicated by (^∗^) and italic font.

**Table 6:** The relationship between KIR genes and non-AIDS associated comorbid conditions.

KIR genes	Diabetes		Hyperlipidemia		Hypertension		CVD	
	OR (95% CI)	*p* value	OR (95% CI)	*p* value	OR (95% CI)	*p* value	OR (95% CI)	*p* value
Inhibitory *KIRs*								
KIR2DL1	1	—	0.3 (0.01-4.8)	0.362	0.1 (0.01-1.78)	0.129	0.08 (0.01-0.69)	0.022
KIR2DL2	1.1 (0.19-6.65)	0.886	0.5 (0.16-1.4)	0.179	1.3 (0.4-4.29)	0.664	1.04 (0.25-4.31)	0.954
KIR2DL3	1	—	0.7 (0.12-4.29)	0.721	0.08 (0.006-0.99)	0.05	0.2 (0.03-1.46)	0.113
KIR2DL5	2.3 (0.3-17.48)	0.422	1.3 (0.42-3.97)	0.651	1.9 (0.51-7.12)	0.341	0.6 (0.13-2.6)	0.476
KIR3DL1	0.14 (0.01-2.05)	0.151	0.4 (0.08-2.06)	0.272	0.6 (0.1-3.01)	0.493	1	—
Activating *KIRs*								
KIR2DS1	2.1 (0.32-13.7)	0.44	1.4 (0.49-3.8)	0.555	2.7 (0.76-9.35)	0.124	0.8 (0.2-3.29)	0.759
KIR2DS2	1.1 (0.19-6.64)	0.886	0.5 (0.16-1.4)	0.179	1.3 (0.4-4.29)	0.664	1.04 (0.25-4.31)	0.954
KIR2DS3	2.4 (0.39-15.42)	0.34	1.2 (0.42-3.54)	0.715	1.6 (0.43-5.68)	0.501	0.2 (0.02-1.83)	0.11
KIR2DS4	0.14 (0.01-2.05)	0.151	0.4 (0.08-2.06)	0.272	0.6 (0.1-3.01)	0.493	1	—
KIR2DS5	2.3 (0.36-14.35)	0.377	1.03 (0.37-2.85)	0.962	1.8 (0.53-5.75)	0.355	1.04 (0.25-4.26)	0.962
KIR3DS1	2.5 (0.39-15.45)	0.34	1.7 (0.6-4.83)	0.32	2.6 (0.75-9.15)	0.131	0.6 (0.14-2.67)	0.507
*Pseudogenes*								
KIR2DP1	1	—	0.3 (0.01-4.77)	0.362	0.14 (0.01-1.78)	0.129	0.08 (0.01-0.69)	0.022

CVD: cardiovascular disease; OR: odds ratio; 95% CI: 95% confidence interval. We adjusted for age, sex, and family history of comorbid condition. For hypertension and CVD, we also adjusted for smoking and alcohol use. We applied Bonferroni correction, and thus, *p* values of <0.004 were considered significant.

**Table 7:** KIR cluster analysis.

	With diabetes (*N* = 9)	Without diabetes (*N* = 28)	*p* value	With hyperlipidemia (*N* = 31)	Without hyperlipidemia (*N* = 45)	*p* value	With CVD (*N* = 10)	Without CVD (*N* = 92)	*p* value	With hypertension (*N* = 31)	Without hypertension (*N* = 34)	*p* value
Cluster 1^a^												
Yes	6 (37.5%)	10 (62.5%)	0.136	13 (41.9%)	18 (58.1%)	1	3 (8.1%)	34 (91.9%)	0.744	14 (53.8%)	12 (46.2%)	0.456
No	3 (14.3%)	18 (85.7%)		18 (40%)	27 (60%)		7 (10.8%)	58 (89.2%)		17 (43.6%)	22 (56.4%)	
Cluster 2												
Yes	5 (25%)	15 (75%)	1	14 (35%)	26 (65%)	0.352	5 (9.4%)	48 (90.6%)	1	17 (53.1%)	15 (46.9%)	0.46
No	4 (23.5%)	13 (76.5%)		17 (47.2%)	19 (52.8%)		5 (10.2%)	44 (89.2%)		14 (42.4%)	19 (57.6%)	
Cluster 3												
Yes	4 (33.3%)	8 (66.7%)	0.432	11 (42.3%)	15 (57.7%)	1	1 (2.9%)	33 (97.1%)	0.159	11 (55%)	9 (45%)	0.591
No	5 (20%)	20 (80%)		20 (40%)	30 (60%)		9 (13.2%)	59 (86.8%)		20 (44.4%)	25 (55.6%)	
Cluster 4												
Yes	6 (18.2%)	27 (81.8%)	*0.038* ^∗^	26 (38.2%)	42 (61.8%)	0.259	10 (10.9%)	82 (89.1%)	0.592	26 (45.6%)	31 (54.4%)	0.463
No	3 (75%)	1 (25%)		5 (62.5%)	3 (37.5%)		0 (0%)	10 (100%)		5 (62.5%)	3 (37.5%)	
Cluster 5												
Yes	9 (26.5%)	25 (73.5%)	0.562	28 (41.2%)	40 (58.8%)	1	8 (8.8%)	83 (91.2%)	0.294	25 (43.1%)	33 (56.9%)	*0.047* ^∗^
No	0 (0%)	3 (100%)		3 (37.5%)	5 (62.5%)		2 (18.2%)	9 (81.8%)		6 (85.7%)	1 (14.3%)	
Centromeric cluster												
Yes	4 (33.3%)	8 (66.7%)	0.432	9 (37.5%)	15 (62.5%)	0.804	1 (10%)	31 (33.7%)	0.165	10 (52.6%)	9 (47.4%)	0.758
No	5 (20%)	20 (80%)		22 (42.3%)	30 (57.7%)		9 (8.8%)	61 (59.8%)		21 (45.6%)	25 (54.4%)	

CVD: cardiovascular disease. ^a^Cluster 1 is the same as telomeric cluster. Significant *p* values (*p* < 0.05) of Fisher's exact test are indicated by (^∗^) and italic font.

**Table 8:** The relationship between KIR clusters and non-AIDS associated comorbid conditions.

	Diabetes		Hyperlipidemia		Hypertension		CVD	
	OR (95% CI)	*p* value	OR (95% CI)	*p* value	OR (95% CI)	*p* value	OR (95% CI)	*p* value
*KIR* clusters								
Cluster 1^a^	2.7 (0.44-16.45)	0.283	1.27 (0.45-3.58)	0.649	1.7 (0.52-5.82)	0.373	0.8 (0.18-3.6)	0.786
Cluster 2	1.14 (0.19-6.65)	0.886	0.5 (0.16-1.4)	0.179	1.3 (0.4-4.29)	0.664	1.04 (0.25-4.31)	0.954
Cluster 3	2.4 (0.39-15.53)	0.34	1.2 (0.41-3.54)	0.715	1.6 (0.43-5.68)	0.501	0.2 (0.02-1.83)	0.154
Cluster 4	0.14 (0.01-2.04)	0.151	0.4 (0.08-2.06)	0.272	0.6 (0.1-3)	0.493	1 -	—
Cluster 5	1 -	—	0.7 (0.12-4.29)	0.721	0.08 (0.006-0.99)	0.05	0.2 (0.03-1.46)	0.113
Centromeric cluster	2.4 (0.39-15.43)	0.34	0.9 (0.29-2.63)	0.82	1.2 (0.32-4.37)	0.811	0.2 (0.02-2.13)	0.195

CVD: cardiovascular disease; OR: odds ratio; 95% CI: 95% confidence interval. ^a^Cluster 1 is the same as telomeric cluster. We adjusted for age, sex, and family history of comorbid condition. For hypertension and CVD, we also adjusted for smoking and alcohol use. We applied Bonferroni correction, and thus, *p* values of <0.004 were considered significant.

## Data Availability

The clinical, biological, and demographic data used to support the findings of this study are included in the article.
